# Study protocol: perinatal mood treatment study

**DOI:** 10.1186/s13063-024-08086-w

**Published:** 2024-08-06

**Authors:** Kate Wolitzky-Taylor, Misty C. Richards, Amelia Welborn, Vanessa McDonald, Inna Arnaudova, Scott Fears, Heather O’Mahen, Jill M. Newby, Mike Millard, Allison V. Metts, Alan Stein, Nelson Freimer, Michelle G. Craske

**Affiliations:** University of CA – Los Angeles, 405 Hilgard Ave., Los Angeles, CA 90025 USA

**Keywords:** Perinatal depression, Tracking, Evidence-based treatment, Online therapy

## Abstract

Perinatal depression (PND) affects up to 20% of women and is associated with significant impairment and disability in affected women. In addition, perinatal depression is associated with broader public health and multigenerational consequences. Innovative approaches are needed to reduce the burden of perinatal depression through identification, tracking, and treatment of depressive symptoms during the perinatal period. This study is a randomized clinical trial comparing the relative efficacy of a multi-tiered system of care, Screening and Treatment of Anxiety and Depression (STAND) to perinatal care delivered by a reproductive psychiatrist in reducing symptoms of depression and anxiety. A sample of 167 individuals was randomized between week 28 of pregnancy and 6 months postpartum. A secondary aim compares the original online therapy intervention used in the first half of the study to a newer online therapy program used in the second half of the study for individuals assigned to the STAND treatment. The study measures, intervention groups, and analysis methods are described, as well as expected implications. The findings from this study may improve the methods for tracking symptom changes over time, monitoring treatment response, and providing personalized care for individuals with PND. As such, this study may improve the lives of patients with PND and their families and lower the related health care costs to society.

**Trial registration** NCT: 9/24/2021

NCT direct link: https://www.clinicaltrials.gov/study/NCT05056454?term=NCT05056454&rank=1&a=1.

## Introduction

Perinatal depression (PND) is defined as clinically severe depression and anxiety occurring during pregnancy and up to 1 year postpartum that affects up to 20% of women [1, 2]. Depression during pregnancy has been linked to adverse outcomes including poor nutrition, increased substance use, pre-eclampsia, pre-term delivery, and low birthweight, as well as continuation of depressive symptoms postpartum with risk of suicide [3–5]. Death by suicide is a leading cause of maternal mortality, estimated to be 1.6 to 4.5 per 100,000 live births in the USA [6] and 1.27 to 3.7 in countries including the UK, Canada, and Sweden [7–9]. More than 54% of those with postpartum depression report depressive symptoms and anxiety beginning during pregnancy [10, 11]. Postpartum depression is emotionally and physically debilitating during one of the most vulnerable life transitions, compromising personal, household, and social functioning [12].

There are far-reaching public health consequences of untreated perinatal depression. When following the mother-child dyad from pregnancy through 5 years postpartum, the estimated societal cost of untreated perinatal depression in the USA was $14.2 billion for all births in 2017 [13]. The most substantial costs come from productivity losses and maternal health expenditures. Untreated perinatal depression has multigenerational consequences. Children of depressed mothers show greater vulnerability to psychopathology, particularly to depression, anxiety disorders, and behavioral problems (e.g., maladaptive social behavior; hyperactivity; conduct problems), and this is thought to be mediated by the quality of the parent-infant relationship ([14, 15]). They also are at greater risk for poorer cognitive and academic performance [16]. Given the prevalence, impairment, and societal burden of perinatal depression, investigation of effective treatments is crucial.

### Treatment for perinatal depression

Although psychopharmacological effectiveness trials in perinatal populations are limited in number and the results are mixed [17], there remains general consensus supporting the use of SSRIs to treat depression during the perinatal period [18]. Sertraline is considered the SSRI of choice for treating depression while breastfeeding due to its relatively short half-life, low or undetectable infant serum concentrations, and effective metabolism in both mother and infant [19].

The most empirically supported non-pharmacologic treatments for perinatal depression are cognitive behavioral therapy (CBT) and Interpersonal Therapy (IPT), at least in middle- to high-income and non-minority samples [17]. In a meta-analysis that included 73 studies focused on the treatment of perinatal depression, the randomized controlled trials examining the effectiveness of CBT or IPT demonstrated a significant benefit of CBT or IPT over the control condition regardless of modality (i.e., remote, in-person, telephone) and duration (i.e., 6–12 sessions) [17].

The Screening and Treatment for Anxiety and Depression (STAND) system of care is a technology-assisted, scalable approach to increase access to evidence-based mental health care with CBT at its core. STAND is a novel, integrated tiered treatment system that provides patient-centered care by triaging to level of care (monitoring only, digital therapy with coaches, digital therapy assisted by clinicians in training, and clinical care) and then continuously monitors symptoms to adapt level of care. More details about the STAND system of care and its effectiveness can be found in Wolitzky-Taylor et al. [20]. This approach to treatment aims to provide more tailored care to the wide variety of clinical presentations of PND in a readily accessible way.

### Access to care for perinatal depression

The US Preventive Services Task Force recommends mental health screening for all pregnant women up until at least 1 year postpartum. Evidence shows that screening women for depression during the perinatal period may reduce depressive symptoms and reduce the overall prevalence of depression in a given population. Among pregnant and postpartum women, 6 trials (*n* = 11,869) showed 18 to 59% relative reductions with screening programs in the risk of depression at follow-up (3–5 months) after participation in programs involving depression screening, with or without additional treatment, compared with treatment as usual [21]. Yet barriers remain that impede access to mental health care, including a national shortage of providers trained in perinatal mental health care delivery, lack of Medicaid coverage, and structural inequities [22, 23]. As a consequence, despite recent policy changes and screening mandates, PND continues to be underdiagnosed [24], with relatively poor follow-up. Notably, the perinatal period presents unique challenges for women to attend therapy appointments. Indeed, help-seeking is low in this population, with estimates ranging from 15 to 40% [25, 26]. Importantly, racial and ethnic disparities further impact diagnosis, treatment, and ongoing management, with white mothers having higher rates of care for PND compared to their Hispanic and Black counterparts [27]. Online psychotherapy may overcome some of the barriers to attendance through the convenience and relative anonymity of flexibly scheduled therapy material to pregnant women and new mothers in their homes. A small but growing body of research reveals moderate effects of internet-based CBT for anxiety and depression in perinatal samples [28].

### The present study

The present study is designed to address the treatment challenges for PND by comparing STAND, which includes online therapy with coaching for women with moderate depression and clinician-delivered psychological care with psychiatric care as needed for women with severe depression or suicidality, to augmented perinatal psychiatry care (PCC). The primary aim of the study is to evaluate the efficacy of STAND in reducing symptoms of depression and anxiety for perinatal women. This study is currently underway.

## Methods

### Trial registration

The study is registered at ClinicalTrials.gov, NCT05056454.

### Study site

Participants are recruited through UCLA Obstetrics & Gynecology (OB-GYN). Clinical services in this protocol are provided through the UCLA Depression Grand Challenge (DGC) Clinic, a research and training clinic that is housed within the UCLA Depression Grand Challenge program at the University of California, Los Angeles.

### Research operations team

This study is supported by research and clinical staff. The research coordinator is responsible for participant screening, enrollment, and communication, as well as data collection and management. The project manager is responsible for protocol implementation, management of IRB submissions and study documents, database maintenance, data quality control, monthly data monitoring, and study reporting. The study database is built and maintained by informatics specialists, who are also responsible for the development and management of the website that hosts online coaching lessons for this protocol.

### Clinical team

Clinical staff supporting this study includes care providers—coaches, psychiatry fellows, and psychology trainees—who are trained on the provision of service under this protocol for their respective scope. Supervision of the care providers is provided by licensed clinicians including clinical psychologists, psychiatrists, and social workers. Clinical supervision is provided on a weekly basis. The clinical team meets with the principal investigators and research team bi-weekly (1 × /2 weeks) to discuss patient care, protocol adherence, and clinical concerns for active participants. The clinical director is responsible for overseeing the clinic operations, and ensuring clinical care is adhering to study protocol and best practices. Clinical scheduling, communication, and documentation are supported by a clinical care coordinator. Clinical documentation is transferred into research records through the support of a medical scribe.

### Trial committee

The Trial Steering Committee for this protocol is comprised of the directors of the Depression Grand Challenge, principal investigators of this protocol, and the clinical director of the STAND program. This group meets bi-weekly (1 × /2 weeks) with the research program manager, and research coordinator to review study progress, data quality, protocol deviations, and participant care.

### Trial monitoring

A Data Monitoring Committee was not considered as this is a low-risk intervention. Protocol deviations are reported to the relevant centers as directed by the UCLA IRB and Office of Human Research Protection Program. Protocol amendments are communicated to clinical and research team members by the program manager through email notifications describing the changes and any impacts to operations. These email notifications include the updated protocol and relevant updated study documents. The clinical trial registry is kept up to date with protocol amendments and trial descriptions as changes are made.

### Study design

The overall aim of the Perinatal Mood Tracking and Treatment study is to examine a scalable, novel intervention approach in comparison to augmented standard psychiatric care. Participants who present with depression as defined by ≥ 11 on the Edinburgh Postnatal Depression Scale (EPDS) are randomized into one of two treatment conditions: (1) Screening and Treatment for Anxiety and Depression (STAND), the integrated tiered treatment system which is the novel approach being tested and includes online therapies with nonprofessional coaches for moderately depressed women and clinician care for severely depressed women^1^ and (2) augmented perinatal psychiatric care (PCC), consisting of medication management and supportive therapy for up to 6 months, delivered by psychiatrists with expertise in reproductive psychiatry. The PCC condition expands upon typical care in that patients are screened for depressive symptoms multiple times throughout the perinatal period by their obstetrician and receive a timely referral and appointment with a reproductive psychiatrist within the OB-GYN Department when appropriate. In addition, patients receive highly specialized mental health treatment that includes both medication management and a detailed list of community referrals ranging from therapists to lactation consultants.

### Study-specific aims

1. Compare the relative efficacy of an integrated, tiered-treatment model (STAND) and PPC. This comparison will be assessed through changes in scores on mental health measures (primary outcomes: depression, anxiety, suicide; secondary outcomes: sleep quality, functional impairment) between baseline and treatment endpoints (i.e., 13 and 26 weeks after baseline).
2. A secondary aim is to evaluate noninferiority of two versions of STAND online therapy across two cohorts within the larger trial described in Aim 1. Specifically, this aim compares two versions of STAND that utilize different online therapies (although both target perinatal depression); the second version replaced the first as part of ongoing improvements to the STAND system of care, which includes the development of online therapies within the UCLA Depression Grand Challenge to improve the patient experience. (Aim 1 will include the version of online therapy as a covariate in statistical modeling). Non-inferiority will be evaluated based on statistical significance of group differences in primary and secondary outcomes.

### Participant recruitment

**Fig. 1:**
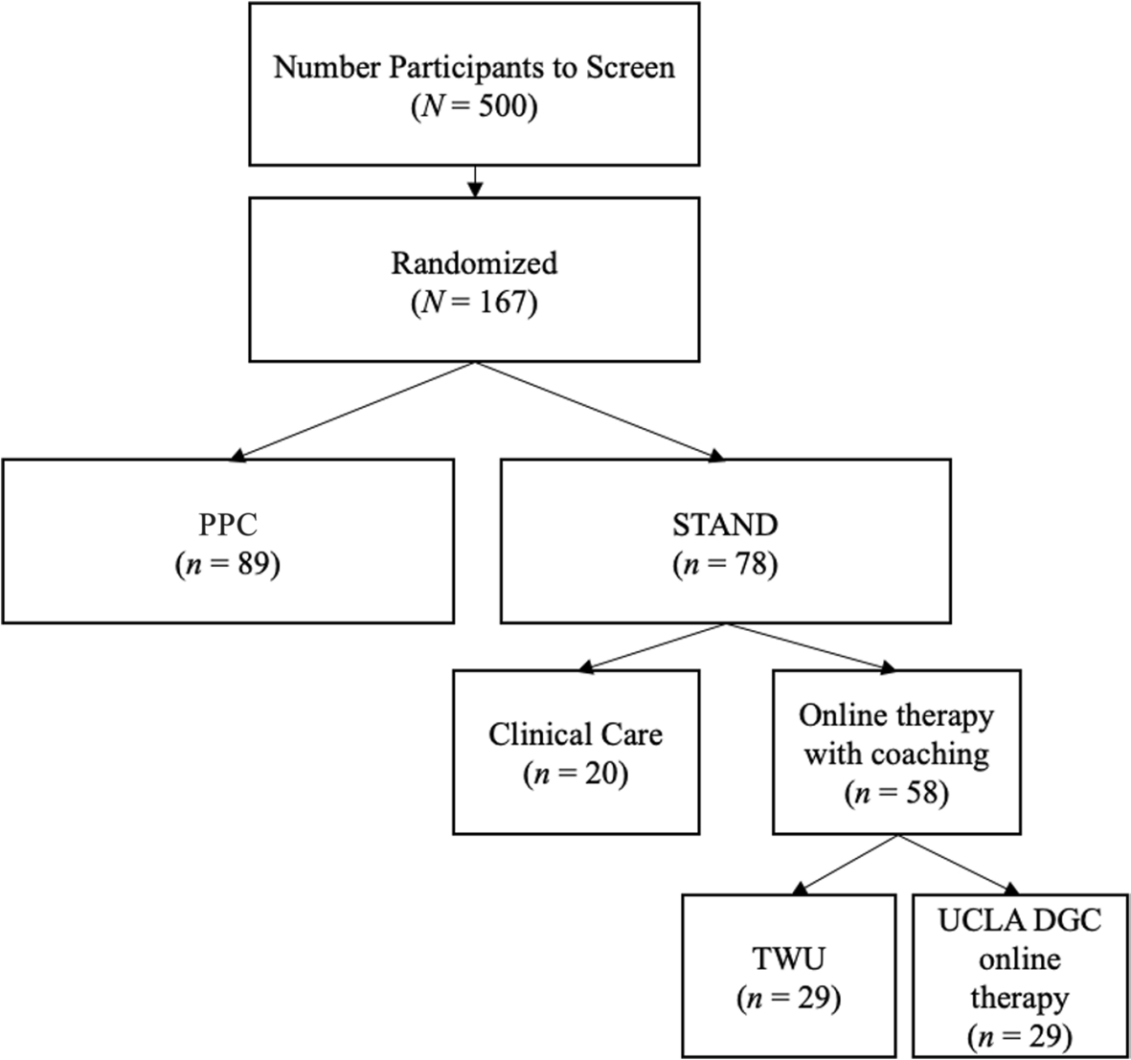
Expected CONSORT flow diagram

The goal is to recruit 167 women who are between week 28 of their pregnancy and 6 months postpartum and are receiving care in clinics in the UCLA Obstetrics & Gynecology (OB-GYN) department. Figure 1 presents a CONSORT diagram that provides the anticipated number of women who will need to be screened to reach out target of 167 participants randomized to either STAND or PPC. Those randomized to STAND will be further split into two cohorts to examine non-inferiority of the two versions of STAND (one including the original online therapy and the second including our own online therapy). Participants are screened by phone by study staff before providing informed consent.

### Inclusion/exclusion criteria

Inclusion requires women to be between 18 and 65 years old, fluent in English, endorse at least moderate depression on the Edinburgh Postnatal Depression Scale (EPDS) (sum ≥ 11) at screening, not currently in individual treatment for a behavioral or emotional problem (e.g., anxiety, depression), between week 28 of a pregnancy to 6 months postpartum, willing to follow study procedures, willing to participate in treatment through the study and follow all study procedures (including provide HIPAA Authorization for research), and have access to the internet via mobile or desktop device.

Exclusion criteria include current treatment by a therapist or a psychiatrist, unstable suicidality (e.g., two or more suicide attempts or self-injurious behaviors resulting in hospitalization in the last 6 months, combined with high ratings on self-reported negative urgency, as assessed via self-report instrument at baseline), current substance use disorder that interferes with treatment (e.g., unable to attend session not under the influence of that substance), current use of cocaine or non-prescribed opioids, principal diagnosis of psychosis unrelated to unipolar or bipolar depression, neurological conditions, severe uncontrolled medical conditions (e.g., anorexia nervosa, cardiac conditions requiring continuous monitoring), and cognitive impairment (e.g., developmental disability, dementia) as identified upon clinical assessment. Care providers under this protocol continuously assess participants for these exclusion criteria throughout their course of care. If any of these criteria are identified, participants are informed they no longer meet eligibility criteria and are withdrawn from the study.

### Random assignment

Participants are randomized to two treatment conditions and following screening and enrollment. The two treatment conditions are (1) augmented perinatal care delivered by a reproductive psychiatrist (PPC) and (2) Screening and Treatment of Anxiety and Depression (STAND). Participants are randomized immediately following consent and are not informed of their treatment condition until after completing the baseline assessment.

### Assessment

#### Screening

Participants identified as potentially eligible for the study after OB-GYN or reproductive psychiatry clinic referral or review of medical records by study staff are contacted and their eligibility for the study determined by phone screening. The screening involves confirming the following information: over 18 years of age, able to read and understand English, a current patient at a UCLA OB-GYN clinic, between approximately 28 weeks of pregnancy and 6 months postpartum, willing to follow study procedures, and not currently in treatment for anxiety or depression. Participants also need a score of 11 or higher on the EPDS (described below) which may be administered during the screening call if one is not available in the medical records. This eligibility process is completed prior to study consent. Referring providers are also aware of the inclusion/exclusion criteria and asked to refer only those patients who may meet those criteria.

#### Baseline

Baseline assessment occurs after enrollment and is offered to potential participants who are between week 28 gestation and 6 months postpartum. Eligible participants complete online self-report assessments of demographics, symptom experience, and personal history (see Measures). Self-report measures of maternal infant bonding and parental bonding^2^ are completed by postpartum participants only (see Table 1 for list of repeated measures). Baseline assessments take an average of 85 min to complete. Participants who complete baseline assessments receive $25.

**Table 1:** Measure administration schedule for repeated measurements

**Measure**	**Abbreviation**	**Time points for measurement**
Medication & Treatment History	N/A	Baseline, 13 weeks, 26 weeks
Screening Assessment for Guiding Evaluation–Self-Report	SAGE-SR	Baseline, 13 weeks, 26 weeks
Maternal Social Support Scale	MSSS	Baseline, 13 weeks, 26 weeks
Edinburgh Postnatal Depression Scale	EPDS	Baseline, 13 weeks, 26 weeks
Pittsburgh Sleep Quality Index	PSQI	Baseline, 13 weeks, 26 weeks
Social Readjustment Rating Scale (Holmes Rahe Stress Scale), Adapted Version	SRRS	Baseline, 13 weeks, 26 weeks
NNDC self-administered Comorbidity Screener	N/A	Baseline, 13 weeks, 26 weeks
Maternal-to-Infant Bonding Scale (post-partum only)	MIBS	Baseline, 13 weeks, 26 weeks
Routines Survey^a^	N/A	Baseline, 13 weeks, 26 weeks
Sheehan Disability Scale	SDS	Baseline, 13 weeks, last clinical appointment, 26 weeks
Computerized Adaptive Testing for Mental Health, perinatal calibration	CAT-MH	TAU (every other week); every week (STAND)
Credibility/Expectancy Questionnaire	CEQ	4 weeks, last clinical appointment

All measures are self-report and administered remotely. Baseline occurs after enrollment, anytime between week 28 gestation and 6 month postpartum. The remaining assessment time points are relative to the date of baseline (e.g., 4 weeks after baseline). The CAT-MH does not include a suicidality assessment for TAU participants

^a^This measure is administered across the UCLA Depression Grand Challenge to get baseline data about personal routines that impact digital tracking data collection and interpretation

Participants complete online self-report assessments 13 weeks and 26 weeks after baseline assessment. Program feedback is also elicited from participants at the end of their participation (26 weeks after baseline assessment). Online surveys at week 13 and week 26 take approximately 55 min each to complete. Participants who complete assessments at week 13 and week 26 receive $35 and $50, respectively.

### Measures

#### Depression screening

Scores on the Edinburgh Postnatal Depression Scale (EPDS) [29, 30], with the suicide item removed, were collected via self-report at three timepoints with their obstetrician, including their intake, 28 week gestation, and 6 week postpartum appointments. A range of elevated EPDS scores has been shown to identify depression (e.g., 10–14; [21, 30]), though a cut-off score of 11 or higher was found to be optimal to meet both DSM-5 and ICD-10 criteria for depression [31]. EPDS scores of 11 or greater were required to be eligible for the current study.

#### Demographics

Several demographic variables are collected at baseline including age, sex at birth, gender identity, transgender status, sexuality, marital status, height, weight, primary and secondary languages, and country of birth.

#### Clinical assessment

The Screening Assessment for Guiding Evaluation-Self-Report (SAGE-SR) is a brief, structured self-report that assesses diagnoses and symptom severity. The SAGE-SR was developed as an alternative to the SCID for use in clinical and research settings and is compatible with the ICD-10 and DSM-5 [32]. The SAGE-SR has demonstrated good to excellent test-retest reliability in a nonclinical sample [33]. The SAGE is used at baseline for descriptive diagnostic purposes, and to assist with screening out exclusionary diagnoses.

#### Measures of mental health outcomes

##### Primary outcome

The perinatal calibration of the Computerized Adaptive Testing for Mental Health (CAT-MH) [34] is the primary outcome measure, providing dimensional severity scores separately for depression, anxiety, mania, and suicidality. Primary assessment periods are baseline, 13 weeks, and 26 weeks post baseline. Because maternal depression over the first six months of the infant’s life is a significant risk factor for infant development [35], a 26-week study protocol period was selected to evaluate maternal outcomes during this sensitive period. Scores at these primary outcome assessment periods will be used to assess symptom outcomes between groups. The CAT-MH is a brief, computerized adaptive test that contains modules for Major Depressive Disorder, Depression, Anxiety, Mania, and Suicidality. The CAT-MH has demonstrated convergent validity in perinatal women [34] and test-retest reliability [36]. Each CAT-MH assessment takes approximately 10 min to complete.

##### Secondary outcomes

The 9-item EPDS, a self-report questionnaire developed to detect postnatal depression, is also administered to measure depressive symptoms at select timepoints [30]. The 9-item EPDS performs similarly to the full EPDS that is both validated and reliable and is used due to concern about the implications of administering EPDS question 10 that asks about suicidality [37].

Functional impairment in family, work, and social domains is assessed using the 3-item Sheehan Disability Scale (SDS) [38]. The SDS has demonstrated high internal consistency and construct validity [39].

Sleep quality is assessed using the Pittsburgh Sleep Quality Index (PSQI; [40]), a 19-item self-report questionnaire that assesses sleep quality and disturbances over a 1-month period. The PSQI has demonstrated strong reliability and validity [41].

#### Credibility, expectancy, and feedback

Treatment credibility and expectancy is assessed 4 weeks after baseline assessment and at the last clinical appointment using the 6-item self-report Credibility/Expectancy Questionnaire (CEQ) [42]. The CEQ has demonstrated excellent internal consistency [42]. Program feedback is also elicited in an online survey in which participants are asked questions regarding their satisfaction with the program, how likely they are to recommend the program, how much time the program assessments took them and their perceptions of the assessment amount, and any changes they would make to the program.

#### Adverse events

In line with protection for human subjects research, adverse events will be reported to the UCLA Institutional Review Board. There is no anticipated harm from trial participation therefore compensation for harm is not provided. Likewise, since there is no anticipated harm, and this study does not involve a new medical device or psychiatric intervention, no formal stopping have been developed.

### Intervention groups

Both treatment conditions consist of care provided through the UCLA DGC research and training clinic. All treatment appointments take place via phone, secure video platform, or in person (depending on patient preference and supervisor consultation) within 6 months of treatment enrollment. If it is recommended for participants to continue receiving care at the time of their study exit, participants are provided with a list of resources and care referrals for community providers.

#### Screening and Treatment of Anxiety and Depression (STAND)

The STAND condition assigns women to either (1) Online therapy with Coaching or (2) Clinical Care, dependent upon baseline CAT-MH scores. Specifically, those with moderate depressive symptoms on the CAT-MH^3^ (≤ 75, and no current suicidality) are allocated to perinatal depression online cognitive behavioral therapy with coaching by nonprofessionals. Those with depression scores in the severe range on the CAT-MH (> 75) or who endorse items on the CAT-MH indicating risk of suicide are assigned to clinical care. Both treatment approaches are described below in detail.

##### Ongoing assessment/measurement-based care

Participants in STAND complete CAT-MH assessments (including suicidality assessment) weekly throughout the 6 months of study participation. CAT-MH scores during treatment and following treatment are an essential part of the STAND system, in that they inform potential adaptations to care and are used to identify and respond to suicide risk, both of which are described below. Research staff routinely monitor the completion of CAT-MH by STAND participants and may contact participants if > 3 consecutive CAT-MH measures are missed. Participants are encouraged to respond to these measures by their providers as well as research staff, with the explanation that these scores are monitored for symptom worsening, and will be used to make decisions about adaptations in care.

##### Adaptation of care

CAT-MH scores throughout the entire 6 months of participation inform treatment switching. Moderately depressed participants assigned originally to online therapy with coaching may be switched to clinical care at any time, during the period of time they are completing the online therapy with coaching (~ 10 weeks) or thereafter, should their depression worsen to the severe level or in the case of suicidality. While in active treatment, two consecutive weeks of a severe depression score on the CAT-MH triggered a symptom worsening alert for digital therapy participants, indicating they should be moved to clinical care. During remote monitoring (following the completion of acute treatment), worsening depression is defined as a CAT-MH depression score that increased 30% increase from prior score at treatment completion and would initiate outreach to re-engage in care if that score exceeded the mild range.

Conversely, severely depressed patients assigned to clinical care can be switched to online therapy with coaching as their depression lessens to moderate or lower levels. Those who complete their clinical care prior to the final 6-month assessment will continue to complete weekly CAT-MH assessments of depression, anxiety, and suicidality, at which times signs of symptom worsening or suicidality will activate re-initiation of either online therapy with coaching or clinical care, depending on symptom severity at that point. Those who require additional care beyond the 6 months are referred to appropriate mental health resources that include therapy and psychiatry in the community.

##### Suicide risk

Treating clinicians and coaches are notified if participants endorse suicidality on the CAT-MH weekly assessment or demonstrate worsening depression. If participant responses to their weekly assessments (CAT-MH with suicide risk items) indicate a risk for suicide, a computer-automated alert is sent to the coach/clinician (if the patient is actively in care), the research team, and a third-party contracted service. The third party contracted service will first make up to three outreach attempts to the patient to assess for and address safety concerns. If the third-party service cannot reach the patient or determines that additional follow-up is needed, the clinical team will make efforts alongside the research team to reach the patient and to address safety needs as clinically indicated. This approach to risk management is implemented for both levels of care within STAND (online therapy with coaching, and clinical care). After triggering a positive suicide risk alert, participants from online therapy with coaching are moved up to clinical care.

##### Online therapy with coaching

Participants with moderate depressive symptoms (i.e., scores ≤ 75 on the CAT-MH) and no current suicidality at baseline who are randomized to the active treatment condition (i.e., STAND) are initially allocated to a perinatal depression online cognitive-behavioral therapy course. Two different online therapy programs will be used at different phases of the study (see Aim 2). For the first 95 participants enrolled in the study, participants in the Online therapy + Coaching arm (*n* = 36) received the perinatal-focused adaptation of This Way Up (TWU), an evidence-based, internet-delivered CBT program for mixed anxiety and depression developed in Australia ([43], see https://thiswayup.org.au/). TWU for the perinatal population is a 3-lesson course called “MUMentum” with six additional optional lessons from TWU for anxiety and depression [44, 45]. Courses in TWU, including “MUMentum” are illustrated lessons that follow a fictional character who experiences mental health difficulties. Throughout the courses, the character learns about the symptoms they experience and how to apply CBT or mindfulness skills. Each individual lesson ends with a summary and homework exercises to be completed prior to the next lesson [45].

The second cohort of participants who are randomized to STAND and assigned to Online therapy + Coaching will receive a new online therapy program for perinatal depression, developed by UCLA DGC and based on the NetMums program, a behavioral activation treatment for perinatal depression [46]. This program includes 10 lessons, with five core modules followed by five additional, optional modules. The psychoeducational and CBT content of each lesson is presented in Table 2. Each lesson includes a homework exercise to reinforce the content of the lesson. Participants are encouraged to practice their lesson homework over the course of the week before the next session. Lessons are completed sequentially and accessed online.

**Table 2:** Content of the UCLA PND online therapy

**Session #**	**Psycho-educational content**	**Intervention content**
1	• The Mood Cycle related to low mood during pregnancy and parenthood • Review of positive emotions	• Review positive activities related and unrelated to pregnancy or baby • Behavioral activation (i.e., practice positive activities)
2	• Introduction to behavioral avoidance	• How to overcome avoidance through alternative coping and activity engagement
3	• Sleep hygiene	• Establish healthy sleep patterns • Create sleep routine • Address environmental factors interfering with sleep
4	• The power of presence and being absorbed in present moment • Review practices linked to positive emotions	• Mindfulness to cultivate present moment awareness • Practice gratitude and generosity to increase positive emotions
5	• Review function, workability, and consequences of worry	• If-Then plans to respond to stress • Opposite action
6	• Review of negative thoughts influencing mood and behavior • Introduce general versus specific thinking	• Practice specific and concrete thinking to address worry and rumination
7	• Review impact of negative thoughts and how to cultivate awareness of thought patterns	• Thought records to identify negative automatic thoughts • Attending to positive information and taking ownership of positive events to increase positive thoughts and emotions
8	• Self-criticism and its link to unhelpful worry	• Self-compassion as an alternative practice to self-criticism
9	• Importance of communication during perinatal period for support purposes • Barriers to effective communication	• Skills for effective interpersonal communication
10	• How noticing positivity can break the low mood cycle • Review of program content and continued practice	• Reliving positive experiences • Relapse prevention and plan for continued practice

In both cohorts (i.e., TWU vs. UCLA DGC online therapy), participants are offered to review material with a STAND coach who answers questions about the digital content, reviews portions of the digital material where appropriate, addresses barriers to home practice completion, and makes home practice recommendations. Coaching sessions are designed to support the lesson after it is reviewed, but lessons can be reviewed during the coaching session if the participant has not had a chance to review it prior to the coaching session. The number of coaching sessions (typically weekly, 45-min sessions) offered matches the number of lessons; 9 for TWU program vs 10 for the UCLA DGC program. Both cohorts are given 10 weeks to complete their lessons and coaching sessions.

Clinical fellows (most of whom are psychology doctoral students) serve as the coaches and receive training in CBT for anxiety and depression, and didactics relevant to the perinatal patient population. They also receive training in how to “coach” (vs. provide therapy) and in the online therapy platform navigation. After training, they receive weekly supervision by licensed clinicians. Formal fidelity ratings will consist of an independent fidelity rater with expertise in cognitive and behavioral therapies for anxiety and depression rating 15% of the sessions (audio recorded) on adherence and competence, using standardized forms adapted from published fidelity ratings forms to meet the needs of assessing fidelity specifically for STAND online therapy.

##### Clinical care

Participants with severe depressive symptoms (CAT-MH ≥ 75) or significant suicidality on the baseline CAT-MH are initially allocated to clinical care, which entails weekly psychotherapy sessions by PhD students in clinical psychology and psychiatry residents, with the goal of 12–16 sessions but more as needed (i.e., if CAT-MH scores remain in the severe range). CAT-MH scores are monitored weekly by clinicians through the STAND dashboard. Psychotherapy is based on a modular treatment approach that is personalized to the patient’s needs. A functional assessment is completed at intake sessions during which clinicians assess the client’s principal problem (most commonly depression given the inclusion criteria, but may also be an anxiety or other disorder). The functional assessment guides selection of an evidence-based intervention suited to the principal problem. Examples of evidence-based interventions include behavioral activation for depressed mood (adapted for perinatal populations in accordance with [47]), cognitive restructuring for excessive worry, and distress tolerance skills for affective instability/self-harm/chronic suicidality (see full list of interventions in Table 3). If the first-line intervention does not result in meaningful clinical change after six sessions per CAT-MH scores (i.e., scores remain in severe range), clinicians revise the functional assessment and shift to a second-line treatment for the principal problem, after consultation with their licensed supervisor. Interventions to address patient non-adherence, acute suicidality, and major life stressors are incorporated as needed. See Wolitzky-Taylor et al. [20] for a more detailed description of the modular, process-based approach to therapy in STAND (Table 4).

**Table 3:** Clinical care treatment approaches for various principal problems

**Problem area**	**Process targeted**	**First line therapy module + medication as appropriate**	**Process being targeted**	**Second line therapy module + medication as appropriate**
Low activity/sadness	Low response contingent positive reinforcement	Pleasant event scheduling (mood monitor, activity schedule, problem solve barriers, sleep schedule for barriers)	Cognitive distortions; rumination	Cognitive restructuring OR mindfulness, acceptance with value driven action; problem solving
Anhedonia	Reward hyposensitivity	Behavioral activation (hedonic and eudaimonic rewards), memory specificity recounting	Reward hyposensitivity	Cognitive restructuring with positive focus; cultivating positivity
Fear/phobia	Deficits in extinction safety learning; avoidance	Exposure therapy	Negative cognitive bias; poor social skills	Cognitive restructuring, mindfulness, acceptance with value driven action; OR social skills training
Worry	Negative cognitive bias	Cognitive restructuring OR mindfulness, acceptance with value driven action	Avoidance (experiential, in vivo)	Exposure therapy; OR mindfulness, acceptance with value driven action; OR social skills training
Sleep dysregulation	Sleep dysregulation	Brief behavioral therapy for insomnia	Negative cognitive bias	Cognitive restructuring
Trauma—fear	Deficits in extinction safety learning; avoidance	Imaginal and in vivo exposure	Negative cognitive bias	Cognitive restructuring in CPT
Trauma—guilt, shame, cognitive distortions	Negative cognitive bias	Trauma narrative with cognitive restructuring and impact statement		
Chronic suicidality, self-harm, affective instability	Low tolerance of distress	Distress tolerance skills in DBT	Poor emotion regulation; interpersonal difficulties	Emotion regulation skills, interpersonal effectiveness in DBT
Mania	Circadian dysregulation	Brief behavioral therapy for insomnia		
Major life stressors (any symptom profile)	Poor coping	Problem solving for controllable stressor; mindfulness, acceptance with value driven action for uncontrollable stressor		
Interpersonal relations (any symptom profile)	Social skills deficits	Interpersonal effectiveness training in DBT

**Table 4:** SPIRIT Checklist

Section/item	Item No.	Manuscript Pg.
Title	1	1
Trial registration	2a	2
	2b	NA
Protocol version	3	NA
Funding	4	1, 25
Roles and responsibilities	5a	1, 25
	5b	1
	5c	NA
	5d	7
Introduction		
Background and rationale	6a	3–6
Objectives	7	6, 8
Trial design	8	8
Study setting	9	15
Eligibility criteria	10	10
Interventions	11a	15–21
	11b	15–16
	11c	18, 20–21
Outcomes	12	13
Participant timeline	13	13, 27
Sample size	14	22
Recruitment	15	9, 11
Allocation:		
Sequence generation	16a	10
Allocation concealment mechanism	16b	11
Implementation	16c	11
Blinding (masking)	17a	11
	17b	
Data collection methods	18a	11–14, 27
Data management	19	6, 8
Statistical methods	20a	23–24
	20b	NA
	20c	NA
Data monitoring	21a	NA
	21b	14
Harms	22	12
Auditing	23	7, 8, 21
Research ethics approval	24	22
Protocol amendments	25	8, 22
Consent or assent	26a	NA
	26b	NA
Confidentiality	27	NA
Declaration of interests	28	25
Access to data	29	25
Ancillary and post-trial care	30	15
Dissemination policy	31a	NA
	31b	NA
	31c	NA
Appendices		
Informed consent materials	32	NA
Biological specimens	33	NA

Psychotropic medications are administered by psychiatry residents and offered as an adjunct treatment when deemed necessary or clinically appropriate during case conference consultation (see below). The schedule of psychiatric care is determined by the provider for up to a maximum of 6 months. Medication prescriptions followed best practices for perinatal populations, typically SSRIs shown to be safe during pregnancy and breastfeeding (e.g., sertraline; [48, 49]).

##### Clinician training

Clinical fellows or psychiatry residents conduct all treatment appointments and receive weekly supervision by licensed clinicians (psychotherapy services) and attending physicians (psychiatric services). Training on psychotherapy interventions involves a once per week (3-h seminar) training series over the course of approximately 3 months. Seminars are led by experts on each psychological intervention option provided in STAND clinical care. Seminars involve didactics on the intervention content and role-plays to ensure understanding. Weekly case conferences are attended by clinical fellows, psychiatry residents, licensed clinicians, and attending physicians during which clinical care and patient needs are discussed, including recommendations for addition of psychotropic medications to the psychological intervention. Informal fidelity checks are done by supervisors listening to sections of recorded sessions, and clinicians are provided with feedback. Formal fidelity ratings will consist of an independent fidelity rater with expertise in cognitive and behavioral therapies for anxiety and depression rating 15% of the sessions (audio recorded) on adherence and competence, using standardized forms adapted from published fidelity rating forms to meet the needs of assessing fidelity specifically for STAND clinical care.

#### Perinatal Psychiatric Care

Patients randomized to PPC received medication management and supportive therapy, and referrals to community resources ranging from therapists to lactation consultants. In general, the PPC protocol consisted of a comprehensive, diagnostic psychiatric assessment that included a history and mental status examination plus medication management (using a medication algorithm based on [48]) where appropriate and supportive therapy using a standardized approach [50]. Medication prescriptions followed best practices for perinatal populations, typically in the form of SSRIs shown to be safe during pregnancy and breastfeeding (e.g., sertraline; [49]). Referrals to longer-term care we provided where appropriate at the final visit. Throughout the treatment, participants complete CAT-MH assessments every other week (without suicidality assessment), but these data are not shared with the treating psychiatrist.

##### Psychiatry training

Psychiatry residents conduct all treatment appointments and receive supervision by attending psychiatrists. Residents are trained by the same reproductive psychiatrist in how to evaluate, diagnose, and treat MDD with peripartum onset, including medication management and provision of supportive psychotherapy [50].

### Sample size and power analysis

#### Aim 1 Power to identify difference in depressive symptom severity between TAU and STAND

To estimate power to detect a statistically significant difference in reduction in depression scores between STAND and PPC, from baseline to 13 and 26 weeks, we used a GLMM regression with the longitudinally assessed dichotomized depression status as the outcome and treatment group as the predictor. An individual-specific random effect was used to account for the intra-individual correlation of depression status. Drop-out rate was simulated to be 25%. We used effect sizes from the literature in terms of comparison between cognitive behavioral therapy and treatment as usual for perinatal depression. Specifically, we evaluated power considering ranges of effect sizes of STAND compared to PPC centered on 0.65 [51], and including the lower and upper limits of the 95% CI of the effect size estimate from Sockol et al. (2015) (0.54–0.76). Based on these estimates, we expected to have sufficient power to detect differences previously reported in the literature in a sample of 120 women. Of note, the intent-to-treat analysis will retain any participants who complete at least the baseline assessment and one lesson/session, thus maximizing statistical power.

#### Aim 2. Power to evaluate equivalency between STAND using TWU versus STAND using UCLA PND online therapy

At the time at which 95 participants were enrolled, a secondary aim was developed to evaluate the equivalency between TWU and UCLA DGC online therapy for perinatal depression. To determine the sample size needed to accommodate this secondary aim, a power analysis was performed using an effect size estimated from the 36 participants who were assigned to online CBT + coaching in the first cohort (using the TWU online program). A linear mixed model regression was used to estimate the effect of time on CAT-MH depression severity for receiving online CBT + coaching. Results indicated a significant effect, whereby CAT-MH depression severity scores reduced by 12 points at each subsequent assessment point (week 13 and week 26) (*β* = − 12.55, *p* < 0.001). We then tested for equivalence of effects of two treatments with a treatment effect size for group 2 equal to group 1. Specifically, we used the identified treatment effect size and sample size from the first cohort (group 1, *n* = 36), and tested sample sizes for a second treatment group ranging from *n* = 5 to 37, and standard errors of treatment effects for both groups ranging from 4 (equal to group 1 standard error) to 10, and equivalence bounds ranging from (−1, 1) to (−10, 10). It was determined that we can show equivalence for bound values above (−5,5) and for sample sizes > 20 provided the standard errors of the effect sizes of the new UCLA DGC online therapy (group 2) are similar to the standard errors of the effect sizes for the TWU online therapy (group 1). As a result of this analysis, we added 72 participants: 36 randomized to STAND, of whom approximately 85% (*n* = 27) are expected to receive online therapy. Of those *n* = 27, approximately 25% (*n* ~ 7) are expected to drop-out, leaving a completer sample of *n* = 20; and 36 randomized to PPC, to arrive at a final sample of *N* = 167.

### Data analysis plan

Intent-to-treat analyses will be performed using multilevel modeling (MLM). The intent-to-treat approach includes all patients who are randomized in the analysis regardless of whether they completed the treatment, yields accurate conclusions regarding intervention effectiveness, and preserves randomization benefits [52]. Multivariate MLM (MMLM) will be used to allow for multiple dependent variables in our models. The primary outcomes MMLM will include major assessment points (baseline, week 13 from baseline, week 26 from baseline) on each of the CAT-MH subscales (depression: CAT-DI, anxiety: CAT-ANX, and suicide: CAT-SS). The secondary outcomes MMLM will include 13-week and 26-week assessments for the EPDS, SDS, and PSQI. MMLM increases power, allows for measures collected at different time points, includes all assessments if at least one of the dependent variables is measured at that assessment, and minimizes type I error inflation [53]. Baseline levels of measures will be included as covariates in all analyses to reduce error variance and to correct for any pretreatment group differences. To address our secondary aim, additional MMLM analyses will be conducted for STAND participants allocated to online therapy + coaching to evaluate the non-inferiority of UCLA DGC online therapy compared to the original online therapy used (TWU), for both primary and secondary outcomes.

Univariate MLMs will be conducted without the multiple dependent variables if this multivariate analysis indicates a difference between groups. We will select the growth curve model that best fits the data in each model. Time will be centered at a post-treatment assessment point so that treatment group differences would test the difference between groups at the end of treatment. We will use the t to d conversion to estimate effect sizes for all significant effects.

### Trial status

The randomized trial of PPC vs. STAND is underway as of May 2021. We anticipate that enrollment will be completed by December 2023. Treatment and the 6 months of study participation will conclude June 2024. The project was approved by the institutional review board at UCLA (IRB #20-001924).

## Discussion

The evaluation of a tiered system of care that matches patients with an appropriate level of evidence-based care for PND has the potential to improve clinical outcomes and prevent more serious consequences. While our study is limited in that formal patient input was not involved in protocol development, we plan to collect patient feedback upon study completion to ensure that future studies benefit from patient experiences. The findings from this study may enable us to develop and implement better ways of tracking symptom changes over time, of monitoring treatment response, and of providing personalized care for the variety of clinical presentations observed in individuals with PND. As such, this study has the potential to improve lives of patients with perinatal depression and their families and lower health care costs to society.

## Data Availability

The final trial dataset will be provided to other research groups upon request.

## Footnotes

1 The typical STAND model of care also includes a third level of care for minimal to mild symptoms. This project uses the levels of care for moderate and severe depression only.

2 If participants endorse aggressive thoughts towards their infant when completing self-report assessments about mother-infant bonding, the research team is notified, and the participant is contacted to assess any risk. Upon completion of the outreach, the participant’s treating OB-GYN and study investigator are also be notified.

3 Note that study inclusion was determined on the basis of EPDS scores ≥ 11, indicating significant perinatal depressive symptoms, whereas scores on the CAT-MH were not used to determine eligibility into the study but were used to make level of care decisions for those randomized to STAND. Thus, although unlikely, a participant could theoretically have exceeded the EPDS cutoff but scored in the normal to mild range on the CAT-MH (scores from 0–64) as opposed to the moderate range (65–74). For the purposes of simplicity, we refer to STAND digital therapy participants as those in the moderate range of depressive symptoms.
